# Association between infant feeding practices and infant growth by maternal HIV and antiretroviral therapy status: a prospective study in Cape Town, South Africa

**DOI:** 10.1186/s12884-026-09249-0

**Published:** 2026-05-21

**Authors:** Janice L. Rhoda, Luke Hannan, Hlengiwe Madlala, Thokozile Malaba, Landon Myer, Andrew Boulle, Mary-Ann Davies, Dorothy C. Nyemba, Emma Kalk

**Affiliations:** 1 Division of Epidemiology and Biostatistics, School of Public Health, University of Cape Town, Falmouth Building, Anzio Road, Western Cape, Western Cape South Africa; 2 Centre for Integrated Data and Epidemiological Research, School of Public Health, University of Cape Town, Cape Town, Western Cape South Africa; 3 Health Intelligence Directorate, Western Cape Government Department of Health & Wellness, Cape Town, Western Cape South Africa; 4 Wits RHI, University of the Witwatersrand, Johannesburg, Gauteng South Africa

**Keywords:** HIV-Exposed Uninfected, HEU, infant growth, feeding practices, weight-for-age, length- for-age, South Africa

## Abstract

**Background:**

Infants who are HIV-Exposed Uninfected (HEU) may experience adverse growth outcomes compared to those HIV-Unexposed Uninfected (HUU). However, there are limited data from the context of universal antiretroviral therapy (ART) and promotion of extended breastfeeding (BF) for infants HEU. We compared the growth of infants HEU and HUU enrolled January 2017-July 2018 from the same community in Cape Town, South Africa.

**Methods:**

Data were collected during pregnancy and at seven days, 10 weeks, six and 12 months postpartum. Infant birth weight and length were converted to weight-for-age and length-for-age z-scores (WAZ, LAZ) using Intergrowth-21st software. We determined WAZ, LAZ and weight-for-length z-scores (WLZ) at postpartum visits using World Health Organization Anthro survey analyzer tool. Linear mixed-effects models were fit to compare WAZ, LAZ and WLZ between infants HEU and HUU and assess associations with infant feeding practices, social support and food security, adjusting for infant HIV exposure status, time (study visit), maternal age, employment and marital/cohabiting status, household food security status and duration of BF.

**Results:**

Among 796 mother-infant pairs, infants HEU (50%) had shorter median BF duration [73 days; interquartile range (IQR) 12-222 vs 209 (IQR 72-365) for infants HUU (*p* < 0.001)]. Complementary feeding was introduced early with a high proportion having sweet beverages and no differences between infants HEU and HUU. Among all infants WAZ increased at 12 months vs 7 days [β = 0.621; 95% CI 0.523 to 0.720] but LAZ decreased [β = -1.260; 95% CI -1.406 to -1.114], with higher WLZ [β = 0.171; 95% CI 0.026 to 0.316] at 12 months vs 10 weeks and no impact of HIV exposure or BF. Infants of mothers married/cohabiting (proxy for social support) had higher WAZ and LAZ. Food security was associated with higher WLZ [β = 0.341; 95% CI: 0.029 to 0.653].

**Conclusions:**

In our study, overall infants experienced suboptimal length growth but gained weight with no difference by HIV status or BF. Being married/cohabiting improved all growth parameters and food security improved growth in weight. Public health interventions should enhance social support and food security to promote optimal growth.

**Supplementary Information:**

The online version contains supplementary material available at https://doi.org/10.1186/s12884-026-09249-0.

## Background

Due to the advancement of vertical transmission prevention of HIV (VTP), policies infants who are HIV-Exposed Uninfected (HEU) comprise a growing population in sub-Saharan Africa (SSA). In 2022, approximately 16 million children globally were HEU, [1] with over 90% living in SSA, 3.5 million (23.8%) in South Africa (SA), which accounted for one of the top five countries globally [2]. Infants who are HEU experience various exposures including to maternal HIV and antiretroviral therapy (ART) *in utero*, via breastfeeding (BF) as well as directly via infant post-exposure prophylaxis (PEP) [2, 3]. Studies have shown that infants HEU are at risk of adverse health effects and poorer growth [4–8], including respiratory infections, diarrhoea-related illnesses and developmental delay, than infants who are HIV-Unexposed Uninfected (HUU), increasing their risk for morbidity and mortality [2].

Studies in SA [5–9] and SSA [8, 10, 11] reported lower weight-for-age z-scores (WAZ) and length-for-age z-scores (LAZ) in infants who are HEU compared to their HUU counterparts. Lower WAZ and LAZ among infants who are HEU may be due to factors such as lower birthweight, lower rates of BF and differences in food security and social support [12] that can impact maternal and child nutrition including complementary feeding practices. *In utero* fetal exposure to HIV and ART has been associated with various adverse birth outcomes including low birthweight (LBW) [13, 14] among infants who were HEU compared to those HUU. LBW together with lower WAZ and LAZ up to 12 [15] and 18 months [8] of age in infants who are HEU suggest that LBW may be on the causal pathway to subsequent lower WAZ and LAZ. In addition, lower birth weight was found to predict undernutrition among infants who were HEU compared to those who were HUU [16].

The benefits of BF are well understood, [17] and breastmilk is recognised as a crucial part of infant health and development, regardless of HIV exposure. Lower rates of BF in infants who were HEU vs. HUU corresponded with poorer growth outcomes for this population [15]. The World Health Organization (WHO) recommends the introduction of complementary feeding to infants from six months old [18]. Early and inappropriate introduction of complementary feeds is common in SA and SAA [15, 19–21] reasons are complex including global shifts in dietary patterns in low- and middle-income countries (LMICs), lack of nutritional knowledge, mothers returning to work, cost of commercially available baby products, concerns about HIV transmission, insufficient milk production and poor growth [20, 21]. Early introduction to complementary feeding has been found to negatively impact growth, with an increased prevalence of obesity as opposed to underweight [22] which can be apparent as early as 12 weeks old [23]. Limited data exist that explore the introduction of complementary feeding by maternal HIV status [24].

The extent to which growth disparities between infants HEU and HUU are mitigated in the context of universal ART and maternal BF is unclear, and differences in complementary feeding practices, food security and social support between infants HEU and HUU may drive ongoing suboptimal growth in infants HEU. We aimed to compare growth parameters between infants who were HEU and HUU from birth to 12 months old and the associations with types of infant feeding practices (BF and complementary feeding) and Household Food Security Status (HFSS) and social support in an era of universal ART in pregnancy policies.

## Methods

### Study setting

B-Positive was a prospective cohort study which enrolled pregnant women attending antenatal care (ANC) at the Gugulethu Midwife Obstetric Unit (GMOU), a primary maternity healthcare facility in Gugulethu, an urban township in Cape Town, SA. The study aimed to comprehensively monitor the effectiveness, impact, and risks of the WHO Option B+ approach for the prevention of vertical transmission of HIV. WHO Option B+ recommended life-long ART for all Women Living with HIV (WLHIV) irrespective of their CD4 count or clinical status. GMOU midwives provide ANC, delivery, and immediate postnatal services for uncomplicated pregnancies not requiring hospital care. Women can be referred to the hospital for further care at any stage of pregnancy according to standard operating procedures [25]. Women were enrolled at their first antenatal visit regardless of whether or not they were subsequently referred to hospital and the cohort included both uncomplicated and high-risk pregnancies. HIV screening is provided regularly throughout pregnancy for women with previous negative or unknown HIV status. The facility serves a population of approximately 350,000 with an antenatal HIV prevalence of 30% in 2015 [26].

### Study design and study participants

**Fig. 1:**
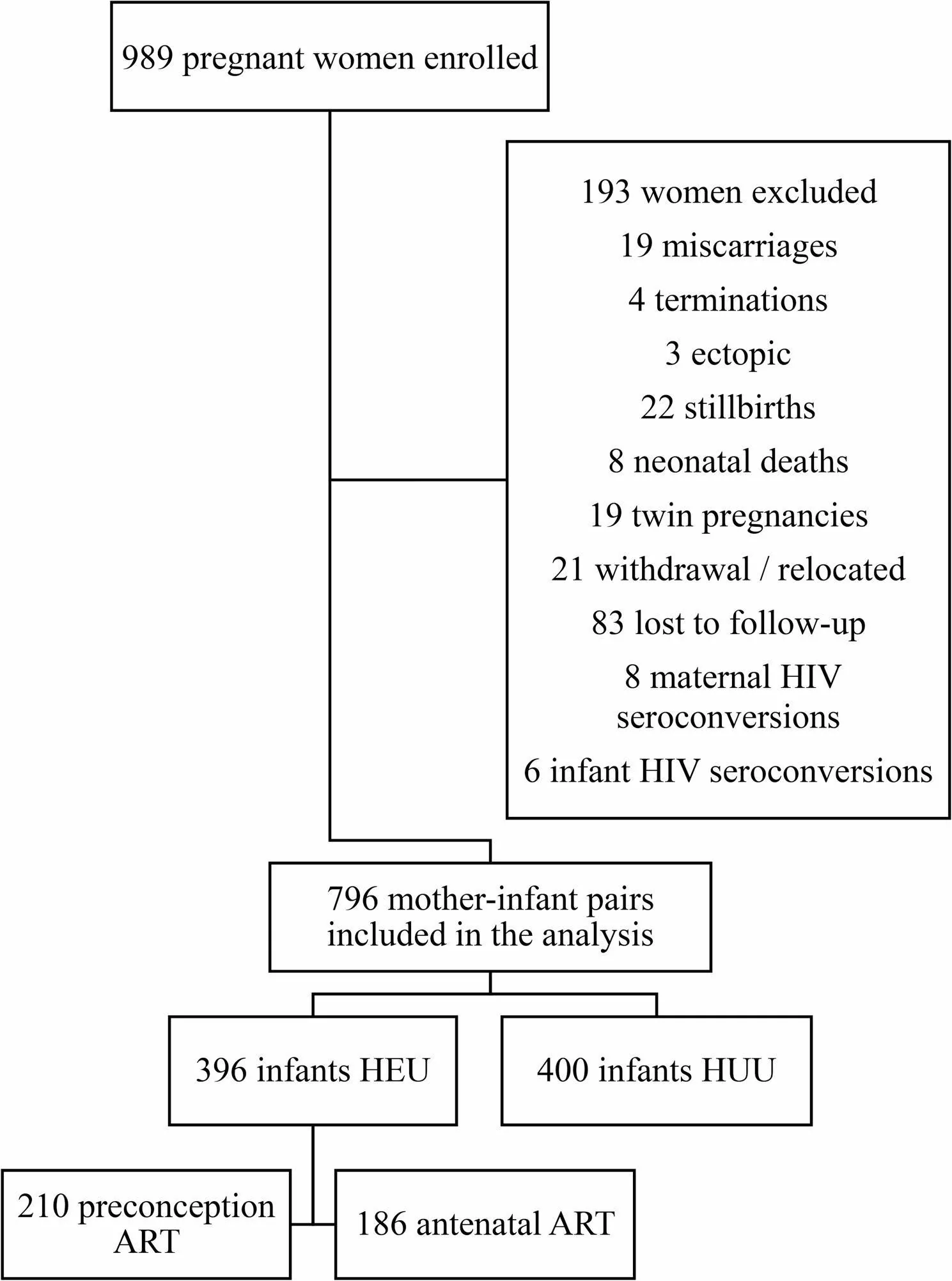
Study flow diagram of participants in a cohort study of pregnant women living with and without HIV and their infants in 2017 to 2018 in Cape Town, South Africa. *HIV* Human Immunodeficiency Virus, *ART* Antiretroviral therapy. *HEU* HIV-Exposed Uninfected, *HUU* HIV-Unexposed Uninfected

Pregnant women aged ≥ 18 years (ratio of 1:1 living with HIV: without HIV) were recruited between January 2017 to July 2018, at their first ANC visit. The women attended between one to three antenatal study visits prior to delivery depending on the gestational age (GA) at enrolment. GA was determined by an ultrasound. After the birth of the baby, mother-infant pairs attended four postnatal visits at seven days, 10 weeks, six and 12 months. Of the 989 women who enrolled in B-positive, 796 mother-infant pairs with live singleton births and birth anthropometry available were included in this analysis (Fig. 1). We included those with singleton live births who attended at least two postnatal visits.

### Sample size consideration

The sample size was determined by the power calculations for the parent study. In this secondary analysis, we performed a power analysis to determine the statistical power for growth disparities between infants who are HEU and HUU. Based on insights from previous research and past studies conducted in SA, we considered that a sample size of 600 provides adequate power (> 80%) to identify absolute differences in growth patterns between infants who were HEU and HUU, with a significance level set at 0.05.

### Study procedures and data collection

Birth weight and length measured by facility staff, as recorded in the South African Road to Health Booklet (RTHB), were abstracted to derive birth WAZ and LAZ to represent surrogate measures of fetal growth. Infant weight and length were measured at each study visit by trained study staff. Infant weight was assessed with precision to the nearest 10 g using a calibrated digital infant scale (MTB 20 Baby Scale, Adam Equipment, Milton Keynes, UK), without clothing and diapers. All infant length measurements were taken while the infant was recumbent with the use of an infant length measuring board. Length measurements were recorded to the nearest 0.25 centimetres using a rigid recumbent length board (ADE MZ 1013 Baby Length Measuring Rod, ADE, Hamburg, Germany). Two measurements of weight and recumbent length were taken at each visit and the average was calculated to ensure accuracy and avoid outliers. There were no big differences between any two measures of weight and length. Medical information was collected from antenatal, obstetric, medical and laboratory records.

Data regarding maternal demographics including whether married/cohabiting, pregnancy history, and healthcare-related information were collected via interviewer-administered questionnaires and recorded using Research Electronic Data Capture (REDCap) [27]. Maternal HIV status and testing results were self-reported and confirmed through medical chart review. Women who tested negative for HIV based on a rapid antibody test were retested up to two times during pregnancy depending on their GA at enrolment as per VTP guidelines. [28] Women who seroconverted during the study were excluded to avoid exposure misclassification. HIV Polymerase Chain Reaction (PCR) testing of infants who were HEU was undertaken at birth and six to 10 weeks according to national guidelines. [28] Infants who acquired HIV any time during follow-up were excluded. To assess infant feeding practices, including introduction to complementary feeding, standardised questionnaires were used based on 24-hour recall whereby mothers reported their infant’s feeding (breastmilk, complementary foods) since the previous morning. Variables related to the types of complementary feeding by age six months were defined based on the Guidelines of the WHO Global Strategy for Infant Feeding recommendations: solid, semi-solid or soft foods; egg and/or flesh food; sweet beverages; and vegetables and/or fruit. [18] The Alcohol Use Disorders Identification Test (AUDIT) [29] was used to collect alcohol related information to measure alcohol intake during pregnancy. The Six-Item Short Form of the Food Security Survey Module (HFSS) was used to identify households with food insecurity and those with very low food security. [30] It is a validated measure of household food insecurity widely used in Africa and SA. [31] Socio-economic status (SES) was determined using an asset score (Table 1).

### Exposure and outcome

For this secondary analysis, data collected at the enrolment and postpartum visits were included. The primary exposure was infant feeding practices, including BF. Foods categorized as solid, semi-solid, or soft foods included: dairy products (yoghurt, cheese, or cream); cereals, porridge, bread; eggs; fruit or vegetables; and meats or fish. Sweet beverages comprised any fruit juice or fizzy drink intake. The primary outcome was infant growth up to 12 months old using WAZ and LAZ. The birth z-scores were produced using Intergrowth-21st software [32] which adjusted for GA at birth and infant sex. WHO Anthro Survey Analyser tool [33] was used to generate WAZ, LAZ and weight-for-length z-scores (WLZ) at 10 weeks, six and 12 months.

Prevalence of malnutrition, comprising undernutrition (stunting [LAZ < -2 SD], wasting [WLZ < -2 SD], underweight [WAZ < -2 SD]) and overnutrition (overweight [WLZ > + 2 SD]) was determined. [33, 34] Stunting results from chronic undernutrition, which impairs linear growth while wasting occurs due to a shorter period of inadequate nutrition. Underweight may arise from stunting, wasting, or a combination of both. Conversely, overweight and obesity occur when weight is excessive in relation to height, resulting in an imbalance between energy intake (excessive) and energy expenditure (insufficient). [35] Z-scores > 3 or < -3 were reviewed and corrected in the case of data capture errors. Unexplained values > 5 for WAZ and WLZ, > 6 for LAZ or < -6 for WAZ and LAZ and < -5 for WLZ were excluded from the analysis. These scores accounted for < 3.5% of all measurements.

### Statistical analysis

Data were exported from REDCap into the R statistical package (version 4.1.1) for analysis. Descriptive statistics were used to describe maternal and infant characteristics, infant feeding practices, age at introduction to complementary feeding, and z-scores at each age. *P*-values for categorical data were calculated using either Pearson’s chi-squared test, Fisher’s exact test or Cochran-Armitage test as appropriate. *P*-values for means were calculated using Student’s t-test and for medians the Wilcoxon rank-sum test was used to assess differences in the distribution between groups. The lme4 package in R was used to conduct a mixed-effects longitudinal analysis. Linear mixed-effects (LME) models were fitted separately for WAZ, LAZ and WLZ entered as continuous dependent variables. Each model included a random effect for each subject, with explanatory variables including infant HIV exposure status, time (study visit), maternal age, employment status, HFSS and marital/cohabiting status. To examine the linearity of maternal age in relation to WAZ, LAZ, and WLZ, we fitted LME models with splines. However, as we did not identify any significant deviations from linearity for maternal age it was grouped into five categories. Birth weight and GA were not included as they may be co-linear with infant HIV exposure and infant growth, lying on the causal pathway between these variables, [36–38] although this was not assessed. Infant feeding variables including any BF, duration of BF and exlcusive breastfeeding (EBF) up to six months old were entered into separate models as fixed effects to avoid multicollinearity. We present the analysis for LME models with duration of BF. The results of the sensitivity analyses are included as Supplementary Data. All covariates for the LME models were selected a priori.

### Ethical considerations

This study received ethical approval from the University of Cape Town’s Faculty of Health Sciences Human Research Ethics Committee (UCT-HREC-665/2022 and 749/2015). Written informed consent was obtained from women antenatally, and again for themselves and their infants after birth.

## Results

Of the 796 mother-infant pairs, 396 (50%) were HEU and 400 (50%) were HUU by study design (Table 1 Fig. 1). WLHIV were older [31 years; IQR 27–35] than women without HIV [27 years; IQR 23–32 (*p* < 0.001)]; and more likely to be multigravida [median 3 pregnancies; IQR 2–3 versus 2; IQR 1–3 (*p* < 0.001)]. There were no significant differences between WLHIV and women without HIV regarding marital/cohabiting and employment status, SES, and alcohol use during pregnancy. Female infants who were HEU had a statistically significantly lower birth weight, length, and head circumference than those who were HUU. Preterm birth (PTB; gestational age at delivery < 37 completed weeks) was more common in infants who were HEU (15% versus 10%; *p* = 0.02). No significant differences were found between infants who were HEU and those HUU regarding infant sex, delivery mode, small for gestational age (SGA), LBW (birthweight < 2500g), and birth WAZ and LAZ. In addition, mothers of infants who were HEU experienced similar proportions of household food insecurity in the last 12 months compared to mothers of infants who were HUU (Table 1). Maternal demographics and birth anthropometry by maternal ART status in WLHIV are shown in (Table 2). Of the 396 infants who were HEU, 210 (53%) were exposed to both HIV and ART from the time of conception, while 186 (47%) were exposed to ART after conception, but before birth (Fig. 1). WLHIV on ART from conception were older [33 years; IQR 29–37] than women who were exposed to ART after conception [29 years; IQR 26–33 (*p* < 0.001)]. Proportions with household food insecurity in the last 12 months were similar (Table 2).

**Table 1:** Mother-infant pair characteristics and household characteristics by HIV exposure status

	**All women and infants** ***N*** **= 796**	**Women living without HIV and infants HUU** ***N*** **= 400 (50%)**	**Women living with HIV and infants HEU** ***N*** **= 396 (50%)**	***p*** **-value**
**Maternal characteristics **				
**Age (years), median (IQR)**	29 (25-34)	27 (23-32)	31 (27-35)	< 0.001
**Level of education completed n (%)**				
Primary school	30 (4)	11 (3)	19 (5)	0.02
Secondary school	749 (94)	376 (94)	373 (94)	
Tertiary school	16 (2)	12 (3)	4 (1)	
* Missing *	1 (0.1)	1 (0.3)	0 (0)	
**Marital/cohabiting status n (%)**				
Married/ Cohabiting	352 (44)	175 (44)	177 (45)	0.78
Not married/ Not cohabiting	404 (51)	205 (51)	199 (50)	
* Missing*	40 (5)	20 (5)	20 (5)	
**Employment status n (%)**				
Formal employment	267 (33)	121 (30)	146 (37)	0.26
Informal employment	4 (0.5)	3 (1)	1 (0.2)	
Attending school/ College	63 (8)	46 (11)	17 (4)	
Otherworking/ Studying	2 (0.2)	1 (0.2)	1 (0.2)	
Not employed/ Not studying	460 (58)	229 (57)	231 (58)	
Socio-economic status* n (%)				
Lowest SES	287 (36)	137 (34)	150 (38)	0.93
Moderate SES	263 (33)	135 (34)	128 (32)	
Highest SES	243 (30)	126 (31)	117 (29)	
* Missing*	3 (0.4)	2 (0.5)	1 (0.2)	
**Alcohol use in this pregnancy n (%)**				
Yes	81 (10)	40 (10)	41 (10)	0.87
No	715 (90)	360 (90)	355 (90)	
**GA at enrolment (weeks) n (%)**				
>= 20 weeks	432 (54)	234 (58)	198 (50)	0.02
< 20 weeks	361 (45)	165 (41)	196 (49)	
* Missing*	3 (0.4)	1 (0.3)	2 (0.5)	
**GA at delivery (weeks), median (IQR)**	39 (38-40)	40 (38-41)	39 (38-40)	0.15
**Gravidity, median (IQR)**	3 (2-3)	2 (1-3)	3 (2-3)	< 0.001
**Parity, median (IQR)**	1 (0-2)	1 (0-2)	2 (1-2)	< 0.001
Household food security status last 12 months** n (%)				
Food secure	65 (8)	34 (8)	31 (8)	0.74
Food insecure	604 (76)	303 (76)	301 (76)	
* Missing *	127 (16)	63 (16)	64 (16)	
**Birth and infant characteristics **				
**Infant sex n (%)**				
Female	407 (51)	199 (50)	208 (53)	0.43
Male	389 (49)	201 (50)	188 (47)	
**Delivery mode n (%)**				
Vaginal	502 (63)	245 (61)	257 (65)	0.80
Scheduled C-section	108 (14)	55 (14)	53 (14)	
Emergency C-section	166 (21)	89 (22)	77 (19)	
Other	20 (2)	11 (3)	9 (2)	
**Preterm delivery (< 37 weeks) n (%)**	97 (12)	38 (10)	59 (15)	0.02
**Birth weight (kg), median (IQR)**				
Female	3.2 (2.9-3.4)	3.2 (2.9-3.5)	3.1 (2.8-3.4)	0.002
Male	3.2 (2.9-3.5)	3.2 (2.9-3.5)	3.1 (2.8-3.5)	0.34
**Birth length (cm), median (IQR)**				
Female	50 (48-52)	50 (49-52)	50 (48-51)	0.03
* Missing*	17	6	11	
Male	50 (48-52)	50 (48-52)	50 (48-52)	0.61
* Missing *	22	12	10	
**Head Circumference (cm),median (IQR)**				
Female	35 (34-36)	36 (34-37)	35 (33-36)	< 0.001
* Missing *	59	32	27	
Male	36 (34-37)	36 (34-37)	36 (34-37)	0.56
* Missing *	64	32	32	
**Small for GA < 10th centile n (%)**	80 (10)	40 (10)	40 (10)	0.96
**Low birth weight < 2.5kg n (%)**	85 (11)	37 (9)	48 (12)	0.19
**Birth z-score, mean (SD)**				
**Weight-for-age**	-0.06 (1.09)	0.01 (1.09)	-0.12 (1.10)	0.09
**Length-for-age**	0.64 (1.71)	0.70 (1.74)	0.57 (1.69)	0.29
* Missing *	39	18	21	
**Head circumference**	1.31 (1.54)	1.44 (1.58)	1.18 (1.49)	0.03
* Missing *	123	64	59	

*IQR* Interquartile range, *SD* Standard deviation, *GA* Gestational age, *HEU* HIV-Exposed Uninfected, *HUU* HIV-Unexposed Uninfected

*SES included; housing type, toilet, water, electricity, refrigerator, landline, television, level of education, employment status. Cut offs used were >= 7.000 high, <= 4.000 low and other medium

**Household food security status measured using U.S. Household Food Security Module: Six-Item Short Form Economic Research Service, USDA September 2012

*P*-values for categorical data were calculated using either Pearson’s chi-squared test, Fisher’s exact test or Cochran-Armitage test as appropriate

*P*-values for means were calculated using Student’s t-test and for medians the Wilcoxon rank-sum test was used to assess differences in the distribution between groups

**Table 2:** Mother-infant pair characteristics and household characteristics in WLHIV by ART exposure status

	All women and infants HEU *N* = 396	Preconception ART *N* = 210 (53%)	Antenatal ART *N* = 186 (47%)	*p*-value
**Maternal characteristics**				
**Age (years), median (IQR)**	31 (27–35)	33 (29–37)	29 (26–33)	< 0.001
**Level of education completed n (%)**				
Primary school	19 (5)	10 (5)	9 (5)	0.66
Secondary school	373 (94)	199 (95)	174 (93)	
Tertiary school	4 (1)	1 (0.4)	3 (2)	
**Marital/cohabiting status n (%)**				
Married/ Cohabiting	177 (45)	101 (48)	76 (41)	0.26
Not married/ Not cohabiting	199 (50)	102 (49)	97 (52)	
* Missing*	20 (5)	7 (3)	13 (7)	
**Employment status n (%)**				
Formal employment	146 (37)	80 (38)	66 (35)	0.84
Informal employment	1 (0.2)	1 (0.5)	0 (0)	
Attending school/ College	17 (4)	8 (4)	9 (5)	
Other working/ studying	1 (0.2)	1 (0.5)	0 (0)	
Not employed/ Not studying	231 (58)	120 (57)	111 (60)	
Socio-economic status* n (%)				
Lowest SES	150 (38)	72 (34)	78 (42)	0.38
Moderate SES	128 (32)	68 (32)	60 (32)	
Highest SES	117 (29)	69 (33)	48 (26)	
* Missing*	1 (0.2)	1 (0.5)	0 (0)	
**Alcohol use in this pregnancy n (%)**				
Yes	41 (10)	16 (8)	25 (13)	0.06
No	355 (90)	194 (92)	161 (87)	
**GA at enrolment (weeks) n (%)**				
>= 20 weeks	198 (50)	97 (46)	101 (54)	0.11
< 20 weeks	196 (49)	112 (53)	84 (45)	
* Missing*	2 (0.5)	1 (0.5)	1 (0.5)	
**GA at delivery (weeks), median (IQR)**	39 (38–40)	39 (39–40)	39 (38–40)	0.37
**Gravidity, median (IQR)**	3 (2–3)	3 (2–4)	3 (2–3)	0.03
**Parity, median (IQR)**	2 (1–2)	2 (1–2)	1 (1–2)	0.06
Household food security status last 12 months** n (%)				
Food secure	31 (8)	15 (7)	16 (9)	0.61
Food insecure	301 (76)	160 (76)	141 (76)	
* Missing*	64 (16)	35 (17)	29 (15)	
**Birth and infant characteristics Infant sex n (%)**				
Female	208 (53)	109 (52)	99 (53)	0.79
Male	188 (47)	101 (48)	87 (47)	
**Delivery mode n (%)**				
Vaginal	257 (65)	139 (66)	118 (63)	0.65
Scheduled C-section	53 (14)	27 (13)	26 (14)	
Emergency C-section	77 (19)	37 (18)	40 (22)	
Other	9 (2)	7 (3)	2 (1)	
**Preterm delivery (< 37 weeks)** **n (%)**	59 (15)	22 (10)	37 (20)	0.009
**Birth weight, median (IQR)**				
Female	3.1 (2.8–3.4)	3.2 (2.9–3.4)	3.1 (2.7–3.4)	0.33
Male	3.1 (2.8–3.5)	3.2 (3.0-3.5)	3.1 (2.8–3.4)	0.13
**Birth length, median (IQR)**				
Female	50 (48–51)	50 (49–52)	50 (47–51)	0.08
* Missing*	11	5	6	
Male	50 (48–52)	50 (48–52)	50 (48–52)	0.62
* Missing*	10	5	5	
**Head Circumference, median (IQR)**				
Female	35 (33–36)	35 (33–36)	35 (34–36)	0.86
* Missing*	27	14	13	
Male	36 (34–37)	35 (34–37)	36 (34–37)	0.97
* Missing*	32	16	16	
**Small for GA < 10th centile n (%)**	40 (10)	20 (10)	20 (11)	0.69
**Low birth weight < 2.5 kg n (%)**	48 (12)	21 (10)	27 (15)	0.17
**Birth z-score, mean (SD)**				
**Weight-for-age**	-0.12 (1.10)	-0.12 (1.05)	-0.13 (1.16)	0.94
**Length-for-age**	0.57 (1.69)	0.59 (1.72)	0.55 (1.65)	0.82
*Missing*	21	10	11	
**Head circumference**	1.18 (1.49)	1.10 (1.44)	1.27 (1.54)	0.30
* Missing*	59	30	29	

*IQR* Interquartile range, *SD* Standard deviation, *GA* Gestational age, *HEU* HIV-Exposed Uninfected, *HUU* HIV-Unexposed Uninfected, *ART* Antiretroviral therapy

*SES included; housing type, toilet, water, electricity, refrigerator, landline, television, level of education, employment status. Cut offs used were > = 7.000 high, <= 4.000 low and other medium

**Household food security status measured using U.S. Household Food Security Module: Six-Item Short Form Economic Research Service, USDA September 2012

*P*-values for categorical data were calculated using either Pearson’s chi-squared test, Fisher’s exact test or Cochran-Armitage test as appropriate

*P*-values for means were calculated using Student’s t-test and for medians the Wilcoxon rank-sum test was used to assess differences in the distribution between groups

Infants who were HEU had a significantly shorter median duration of BF than those HUU [73 days; IQR 12–222 vs. 209 days; IQR 72–365 (*p* < 0.001]) (Table 3) but similar proportions were introduced to complementary feeding by age six months: half were introduced to solid, semi-solid or soft foods; 34% to sweet beverages between six and 12 months; and 79% consumed fruits and/or vegetables between six and 12 months of age (Table 4). No statistically significant differences were observed among variables assessing the introduction of complementary feeding (Table 4). Stunting at birth were not significantly different between groups (Supplementary Table 1, Additional File 1). At 12 months old the prevalence of undernutrition in both groups was low, with stunting being more common than underweight and wasting (10%, 2%, 1%, respectively). Simultaneously, 19% of all infants were overweight, with no significant differences in the proportion of overweight among those who were HEU compared to HUU (Supplementary Table 1, Additional File 1). At 10 weeks of age, the prevalence of underweight was significantly higher among infants who were HEU in comparison to HUU (9% vs. 5%; *p* = 0.02) (Supplementary Table 1, Additional File 1).

**Table 3:** Infant feeding practices from birth to 12 months

	All infants *N* = 796	Infants HUU *N* = 400 (50%)	Infants HEU *N* = 396 (50%)	*p*-value
**Any breastfeeding n (%)**				
Yes	726 (91)	371 (93)	355 (90)	0.12
No	6 (1)	1 (0.2)	5 (1)	
*Missing*	64 (8)	28 (7)	36 (9)	
**Early initiation of breastfeeding n (%)**				
Within 1 h	604 (83)	314 (85)	290 (82)	0.69
After 1 h	39 (6)	19 (5)	20 (5)	
*Missing*	83 (11)	38 (10)	45 (13)	
**Duration of breastfeeding (days), median (IQR)**				
	181	209	73	< 0.001
	(48–365)	(72–365)	(12–222)	
**Breastfeeding, n (%)**				
**10 weeks**				
Yes	553 (76)	317 (85)	236 (67)	< 0.001
No	114 (16)	28 (8)	86 (24)	
*Missing*	59 (8)	26 (7)	33 (9)	
**6 months**				
Yes	379 (52)	236 (64)	143 (41)	< 0.001
No	264 (37)	96 (26)	168 (47)	
*Missing*	83 (11)	39 (10)	44 (12)	
**12 months**				
Yes	253 (35)	171 (46)	82 (23)	< 0.001
No	387 (53)	154 (42)	233 (66)	
*Missing*	86 (12)	46 (12)	40 (11)	
**Exclusive breastfeeding 3 days after birth n (%)**				
Yes	542 (75)	292 (79)	250 (70)	0.002
No	114 (16)	43 (11)	71 (20)	
*Missing*	70 (9)	36 (10)	34 (10)	
**Exclusive breastfeeding up to 6 months n (%)**				
Yes	51 (7)	24 (7)	27 (8)	0.51
No	603 (83)	313 (84)	290 (82)	
*Missing*	72 (10)	34 (9)	38 (10)	

*HEU* HIV-Exposed Uninfected, *HUU* HIV-Unexposed Uninfected, *IQR* Interquartile range, *SD* Standard deviation

*P*-values for categorical data were calculated using Pearson’s chi-squared or Fisher’s exact test

For medians the Wilcoxon rank-sum test was used to assess differences in the distribution between groups

**Table 4:** Introduction to complementary feeding

	All infants *N* = 796	Infants HUU *N* = 400 (50%)	Infants HEU *N* = 396 (50%)	*p*-value
**Introduction to foods n (%)**				
7 days	1 (0.1)	1 (0.3)	0 (0)	0.51
10 weeks	46 (6)	27 (8)	19 (5)	
6 months	281 (39)	146 (39)	135 (38)	
None	195 (27)	92 (25)	103 (29)	
* Missing*	203 (28)	105 (28)	98 (28)	
**Solid, semi-solid or soft foods at 6 months n (%)**				
Yes	380 (52)	203 (55)	177 (50)	0.31
No	220 (30)	108 (29)	112 (31)	
*Missing*	126 (18)	60 (16)	66 (19)	
**Egg and/or flesh food consumption 6–12 months n (%)**				
Yes	509 (70)	261 (70)	248 (70)	0.61
No	115 (16)	62 (17)	53 (15)	
* Missing*	102 (14)	48 (13)	54 (15)	
**Sweet beverage consumption 6–12 months n (%)**				
Yes	243 (34)	128 (35)	115 (32)	0.72
No	373 (51)	191 (51)	182 (52)	
* Missing*	110 (15)	52 (14)	58 (18)	
**Vegetables or fruit consumption 6–12 months n (%)**				
Yes	574 (79)	296 (80)	278 (79)	0.99
No	62 (9)	32 (9)	30 (8)	
* Missing*	90 (12)	43 (11)	47 (13)	

*HEU* HIV-Exposed Uninfected, *HUU* HIV-Unexposed Uninfected

*P*-values for categorical data were calculated using Pearson’s chi-squared or Fisher’s exact test

LME models of mean WAZ, LAZ and WLZ between infants who were HEU and HUU for duration of BF are shown in Table 5. Among all infants at older ages, WAZ increased (β = 0.621; 95% CI 0.523 to 0.720 at 12 months vs. 7 days) but LAZ decreased (β = -1.260; 95% CI -1.406 to -1.114 at 12 months vs. 7 days), with the result that WLZ was 0.171 higher (95% CI 0.026 to 0.316) at 12 months vs. 10 weeks of age. When infants who were stunted at birth were excluded, LAZ remained affected (Supplementary Table 2, Additional File 2). There was no difference in any of the growth parameters by HIV exposure status or duration of BF, any BF or EBF (Supplementary Tables 3 and 4, Additional File 3 and 4, respectively). Univariable analysis shows that WAZ and LAZ among infants HEU were significantly lower compared to infants HUU (Supplementary Table 5, Additional file 5). Infants of mothers who were married/cohabiting compared to those who were not had higher WAZ and LAZ [β = 0.295; 95% CI: 0.122 to 0.467; *p* = < 0.001] and [β = 0.293; 95% CI: 0.109 to 0.477; *p* = 0.002]. Infants of mothers who were food secure had higher WLZ than those who were food insecure [β = 0.341; 95% CI: 0.029 to 0.653; *p* = 0.03], but other growth parameters were similar (Table 5). When HFSS was excluded, marital/cohabiting status had a consistent impact on WAZ and LAZ, while infant HIV exposure status had a statistically significant impact on WAZ and LAZ (Supplementary Table 6, Additional File 6).

**Table 5:** Linear mixed-effects models of WAZ, LAZ and WLZ between infants HEU and HUU for duration of breastfeeding

Model 1: WAZ between infants HEU and HUU for duration of breastfeeding (*N* of observations = 2138)			
Effect	Estimate	(95% CI)	*p*-value
**Visits**			
7 days	Ref		
10 weeks	-0.067	-0.166–0.033	0.19
6 months	0.363	0.263–0.464	< 0.001
12 months	0.621	0.523–0.720	< 0.001
**Infant HIV exposure status**			
Infants HUU	Ref		
Infants HEU	-0.140	-0.320–0.040	0.13
**Maternal age**			
18–23	Ref		
24–29	-0.074	-0.325–0.177	0.56
30–35	-0.309	-0.576 - -0.042	0.02
36–41	-0.203	-0.506–0.101	0.19
42–48	-1.090	-2.110 - -0.076	0.03
**Employment status**			
Not employed/ Not studying	Ref		
Formal employment	0.182	-0.000–0.365	0.05
Informal employment	0.272	-0.875–1.418	0.64
Attending school/ College	-0.037	-0.365–0.291	0.83
Other, work or studying	0.492	-0.971–1.955	0.51
**Marital/cohabiting status**			
Not married/Not cohabiting	Ref		
Married/ Cohabiting	0.295	0.122–0.467	< 0.001
**Duration of breastfeeding**	0.000	-0.000–0.000	0.78
**Household food security status**			
Food insecure	Ref		
Food secure	0.169	-0.112–0.451	0.24

Model 2: LAZ between infants HEU and HUU for duration of breastfeeding (*N* of observations = 2111)			
Effect	Estimate	(95% CI)	*p-*value
**Visits**			
7 days	Ref		
10 weeks	-1.550	-1.697 - -1.400	< 0.001
6 months	-1.380	-1.530 - -1.232	< 0.001
12 months	-1.260	-1.406 - -1.114	< 0.001
**Infant HIV exposure status**			
Infants HUU	Ref		
Infants HEU	-0.145	-0.338–0.048	0.14
**Maternal age**			
18–23	Ref		
24–29	0.095	-0.175–0.365	0.49
30–35	-0.225	-0.512–0.062	0.12
36–41	-0.094	-0.421–0.232	0.57
42–48	-0.770	-1.851–0.311	0.16
**Employment status**			
Not employed/ Not studying	Ref		
Formal employment	-0.005	-0.201–0.191	0.96
Informal employment	0.869	-0.341–2.080	0.16
Attending school/ College	0.219	-0.132–0.569	0.22
Other, work or studying	0.675	-0.916–2.265	0.40
**Marital/cohabiting status**			
Not married/Not cohabiting	Ref		
Married/ Cohabiting	0.293	0.109–0.477	0.002
**Duration of breastfeeding**	**-0.000**	-0.001–0.000	0.57
**Household food security status**			
Food insecure	Ref		
Food secure	-0.072	-0.373–0.230	0.64

Model 3: WLZ between infants HEU and HUU for duration of breastfeeding (*N* of observations = 1525)			
Effect	Estimate	(95% CI)	*p-value*
**Visits**			
10 weeks	Ref		
6 months	0.124	-0.023–0.271	0.10
12 months	0.171	0.026–0.316	0.02
**Infant HIV exposure status**			
Infants HUU	Ref		
Infants HEU	0.025	-0.174–0.224	0.81
**Maternal age**			
18–23	Ref		
24–29	-0.374	-0.653 - -0.095	0.01
30–35	-0.355	-0.650 - -0.059	0.02
36–41	-0.260	-0.595–0.074	0.13
42–48	-0.790	-1.888–0.307	0.16
**Employment status**			
Not employed/ Not studying	Ref		
Formal employment	0.175	-0.027–0.376	0.09
Informal employment	-0.676	-1.894–0.541	0.27
Attending school/ College	-0.193	-0.554–0.167	0.29
Other, work or studying	0.438	-1.257–2.134	0.61
**Marital/cohabiting status**			
Not married/ Not cohabiting	Ref		
Married/ Cohabiting	0.155	-0.034–0.343	0.11
**Duration of breastfeeding**	**0.000**	-0.000–0.001	0.24
**Household food security status**			
Food insecure	Ref		
Food secure	0.341	0.029–0.653	0.03

*LAZ* Length-for-age z-score, *WAZ* Weight-for-age z-score, *WLZ* Weight-for-length z-score, *HEU* HIV-Exposed Uninfected, *HUU* HIV-Unexposed Uninfected

## Discussion

In this study conducted in the present era of universal ART in pregnancy and support for BF, we found that WAZ improved but LAZ deteriorated for all infants, resulting in increased WLZ and a high prevalence of overweight by 12 months of age, with no significant difference in growth outcomes and BF between infants who were HEU compared to those who were HUU. Infants born to married/cohabiting mothers compared to not married/not cohabiting had a significantly higher WAZ and LAZ while being food secure compared to food insecure was also associated with a significantly higher WLZ, but did not impact WAZ or LAZ.

In contrast to findings in other African populations, we did not find evidence for significantly lower growth in infants HEU vs. HUU although there was a trend to lower WAZ and LAZ. [3, 11, 39–41] In SSA lower WAZ [8, 15] and LAZ [8] among infants who were HEU compared to those who were HUU were found at birth and up to 12 [15] and 18 months old [8]. Instead, we found that being married/cohabiting positively impacted growth in weight and length, suggesting that those with the least social vulnerability and a combined income may be able to provide increased availability of better food options to support optimal growth. A recent study in SA [12] has demonstrated the impact of food security as part of a HIV-related syndemic on the health of infants who were HEU compared to HUU. In our study, growth outcomes of WAZ and WLZ in food secure households improved among all infants up to 12 months, however food security did not provide protection for LAZ growth outcomes, suggesting that it is not just the quantity of food but the quality of food that is needed to support growth in length, and that having sufficient food quantity but of sub-optimal nutritional quality may actually result in malnutrition in the form of overweight in a child with poor length growth. The most recent national estimates for overweight among children younger than the age of five years in SA is 13% [42, 43] which was lower than our study (19%) conducted in an urban area. Another study in SA [39] reported similar findings of linear growth faltering being the most common form of undernutrition by 12 months of age while at the same time authors observed a high prevalence of overweight among both infants who were HEU (16%) and HUU (18%). Our findings confirm the double burden of undernutrition with poor length growth and suboptimal nutrition with weight increase relative to length in SA.

WHO and South African guidelines recommend the introduction of complementary foods from six months old, while BF continues. Similar proportions among infants who were HEU compared to HUU were introduced to: solid, semi-solid or soft foods; egg and/or flesh food; sweet beverages; and consumption of vegetables and/or fruit by six months of age. Inappropriate types of complementary foods were prevalent among both infants who were HEU and HUU. Appropriate introduction of complementary foods, especially increased consumption of fruits and vegetables must be promoted while consumption of fruit juice and other sweetened beverages needs to be discouraged to prevent early obesity among infants. The high proportion of infants exposed to sweetened beverages by six months old may have contributed to the observed prevalence of overweight. Obesity in childhood is a predictor of obesity in adulthood making it critical to address this during the early stages of life.

As in other studies from SA, and SSA [8, 15, 39, 44] duration of BF among infants who were HEU was significantly shorter than among those who were HUU. While neither duration of BF, nor any BF or EBF had a significant impact on the growth outcomes of infants, there are several other benefits of BF which should be promoted among mothers irrespective of HIV status.

### Strengths and limitations

Infants who were HEU and HUU were enrolled contemporaneously from the same community which allowed for the comparison of common socio-behavioural and economic characteristics between the two groups as well as temporal comparisons. Robust GA estimates were obtained by antenatal ultrasound which supported birth WAZ and LAZ calculations. Additionally, we were able to assess the timing of ART exposure in WLHIV. The sample size was big; however, it was insufficient to provide estimates on many of the potential associations. As with all prospective cohort studies, a study effect may have occurred, in which participants changed their behaviour due to awareness of being observed, potentially influencing the findings. The findings may not be generalizable to other settings such as rural areas or those with less-well implemented VTP programmes. When doing observational research, it is challenging to determine causal effects due to potential unmeasured confounders, however we measured several covariates that may be associated with both the independent and dependent variables and included these covariates as fixed effects in the LME models. However, the context in which feeding choices are made is complex.

## Conclusions

In this study, we found improvement in WAZ but not LAZ at older ages in all infants, resulting in higher WLZ at 12 months of age and a high proportion of overweight, with no difference by infant HIV exposure or BF status. Since being married/cohabiting improved all growth parameters and being food secure improved growth in weight only, better social support of all mothers and households and improving both quantity and quality of household foods are needed to improve infant growth, irrespective of maternal HIV status.

## Supplementary Information

Supplementary Material 1.

## Data Availability

The datasets used and/or analysed during the current study are available from the corresponding author on reasonable request and HREC approval.

## Abbreviations

ANC: Antenatal Care
ART: Antiretroviral Therapy
AUDIT: Alcohol Use Disorders Identification Test
β: Beta
BF: Breastfeeding
CI: Confidence Interval
EBF: Exclusive Breastfeeding
GA: Gestational age
GMOU: Gugulethu Midwife Obstetric Unit
HEU: HIV-Exposed Uninfected
HFSS: Household Food Security Status
HIV: Human Immunodeficiency Virus
HREC: Human Research Ethics Committee
HUU: HIV-Unexposed Uninfected
IQR: Interquartile range
LAZ: Length-for-age z-score
LMICs: Low- and middle-income countries
LBW: Low Birth Weight
LME: Linear mixed-effects
PCR: Polymerase chain reaction
PEP: Post-exposure Prophylaxis
PTB: Preterm birth
p: P-value
REDCap: Research Electronic Data Capture
RTHB: Road to Health Booklet
SA: South Africa
SD: Standard Deviation
SES: Socio-economic status
SGA: Small for gestational age
SSA: Sub-Saharan Africa
UCT: University of Cape Town
VTP: Vertical Transmission Prevention
WAZ: Weight-for-age z-score
WHO: World Health Organization
WLHIV: Women living with HIV
WLZ: Weight-for-length z-score
