# Supplementary material for: Association between infant feeding practices and infant growth by maternal HIV and antiretroviral therapy status: a prospective study in Cape Town, South Africa
Source: BMC Pregnancy Childbirth. 2026 May 21;26:1100. doi: 10.1186/s12884-026-09249-0 (PMC13632437; doi:10.1186/s12884-026-09249-0)
Supplement: Supplementary file 1 [file 12884_2026_9249_MOESM1_ESM.docx]

| **Supplementary Table 1.** Prevalence of Malnutrition by Infant HIV Exposure Status from Birth up to 12 months | | | | | |
| --- | --- | --- | --- | --- | --- |
|  |  | **Total infants**  **N = 796** | **Infants HUU**  **N = 400 (50%)** | **Infants HEU**  **N = 396 (50%)** |  |
| **Characteristic** | **Indicator** |  | **Frequency (n%)** | **Frequency (n%)** | ***p-*value** |
|  | **birth** |  |  |  |  |
| Stunting  (n=757) | LAZ < -2 | 52 (7) | 22 (6) | 30 (8) | 0.22 |
| *Missing* |  | 39 (5) | 18 (4) | 21 (5) |  |
| Underweight  (n=796) | WAZ < -2 | 30 (4) | 15 (4) | 15 (4) | 0.98 |
| *Missing* |  | 0 (0) | 0 (0) | 0 (0) |  |
|  | **10 weeks** |  |  |  |  |
| Stunting  (n = 636) | LAZ < -2 | 130 (16) | 60 (15) | 70 (18) | 0.25 |
| *Missing* |  | 160 (20) | 78 (20) | 82 (21) |  |
| Underweight (n = 645) | WAZ < -2 | 56 (7) | 20 (5) | 36 (9) | 0.02 |
| *Missing* |  | 151 (20) | 73 (18) | 78 (20) |  |
| Wasting  (n = 618) | WLZ < -2 | 13 (2) | 7 (2) | 6 (2) | 0.81 |
| *Missing* |  | 178 (22) | 88 (22) | 90 (23) |  |
| Overweight (n = 618) | WLZ > +2 | 120 (15) | 66 (17) | 54 (14) | 0.27 |
| *Missing* |  | 178 (22) | 88 (22) | 90 (23) |  |
|  | **6 months** |  |  |  |  |
| Stunting  (n= 613) | LAZ < -2 | 114 (14) | 56 (14) | 58 (15) | 0.57 |
| *Missing* |  | 183 (23) | 84 (21) | 99 (25) |  |
| Underweight  (n= 612) | WAZ < -2 | 24 (3) | 13 (3) | 11 (3) | 0.80 |
| *Missing* |  | 184 (23) | 84 (21) | 100 (25) |  |
| Wasting  (n= 603) | WLZ < -2 | 9 (1) | 7 (2) | 2 (1) | 0.18 |
| *Missing* |  | 193 (24) | 88 (22) | 105 (26) |  |
| Overweight  (n= 603) | WLZ > +2 | 143 (18) | 72 (18) | 71 (18) | 0.70 |
| *Missing* |  | 193 (24) | 88 (22) | 105 (27) |  |
|  | **12 months** |  |  |  |  |
| Stunting  (n= 612) | LAZ < -2 | 79 (10) | 35 (9) | 44 (11) | 0.17 |
| *Missing* |  | 184 (23) | 85 (21) | 99 (25) |  |
| Underweight  (n=609) | WAZ < -2 | 17 (2) | 8 (2) | 9 (2) | 0.71 |
| *Missing* |  | 188 (24) | 87 (22) | 101 (26) |  |
| Wasting  (n= 604) | WLZ < -2 | 9 (1) | 5 (1) | 4 (1) | 1.00 |
| *Missing* |  | 192 (24) | 90 (23) | 102 (26) |  |
| Overweight  (n= 604) | WLZ > +2 | 149 (19) | 83 (21) | 66 (17) | 0.22 |
| *Missing* |  | 192 (24) | 90 (22) | 102 (26) |  |

*LAZ* Length-for-age z-score, *WAZ* Weight-for-age z-score, *WLZ* Weight-for-length z-score,

*HEU* HIV-exposed-uninfected, *HUU* HIV-unexposed uninfected

P-values for categorical data were calculated using Pearson’s chi-squared or Fisher’s exact test

| **Supplementary Table 2. Linear mixed effects model of LAZ between infants HEU and HUU for duration of breastfeeding (excluding infants stunted at birth)** | | | |
| --- | --- | --- | --- |
| **Model 2: LAZ between infants HEU and HUU for duration of breastfeeding (N of observations = 1926)** | | | |
| **Effect** | **Estimate** | **(95% CI)** | ***p-*value** |
| **Visits** |  |  |  |
| 7 days | Ref |  |  |
| 10 weeks | -1.710 | -1.861 - -1.564 | < 0.001 |
| 6 months | -1.530 | -1.679 - -1.381 | < 0.001 |
| 12 months | -1.450 | -1.592 - -1.299 | < 0.001 |
| **Infant HIV exposure status** |  |  |  |
| Infants HUU | Ref |  |  |
| Infants HEU | -0.144 | -0.331 – 0.043 | 0.13 |
| **Maternal age** |  |  |  |
| 18-23 | Ref |  |  |
| 24-29 | 0.020 | -0.242 – 0.282 | 0.88 |
| 30-35 | -0.221 | -0.501 – 0.059 | 0.12 |
| 36-41 | -0.139 | -0.457 – 0.178 | 0.39 |
| 42-48 | -0.862 | -1.858 – 0.134 | 0.09 |
| **Employment status** |  |  |  |
| Not employed/ Not studying | Ref |  |  |
| Formal employment | 0.014 | -0.176 – 0.204 | 0.88 |
| Informal employment | 0.754 | -0.357 – 1.865 | 0.18 |
| Attending school/ College | 0.055 | -0.273 – 0.384 | 0.74 |
| Other, work or studying | 0.536 | -0.932 – 2.004 | 0.47 |
| **Marital/cohabiting status** |  |  |  |
| Not married/Not cohabiting | Ref |  |  |
| Married/ Cohabiting | 0.183 | 0.005 – 0.362 | 0.04 |
| **Duration of breastfeeding** | -0.000 | -0.000 – 0.000 | 0.88 |
| **Household food security status** |  |  |  |
| Food insecure | Ref |  |  |
| Food secure | -0.034 | -0.340 – 0.272 | 0.64 |

*LAZ* Length-for-age z-score

*HEU* HIV-exposed-uninfected, *HUU* HIV-unexposed uninfected

| **Supplementary Table 3. Linear mixed effects models of WAZ, LAZ and WLZ between infants HEU and HUU**  **for any breastfeeding**  **Model 1: WAZ between infants HEU and HUU for any breastfeeding (N of observations = 2138)** | | | |
| --- | --- | --- | --- |
| **Effect** | **Estimate** | **(95% CI)** | ***p-*value** |
| **Visits** |  |  |  |
| 7 days | Ref |  |  |
| 10 weeks | -0.066 | -0.166 – 0.033 | 0.19 |
| 6 months | 0.363 | 0.263 – 0.464 | < 0.001 |
| 12 months | 0.621 | 0.523 – 0.720 | < 0.001 |
| **Infant HIV exposure status** |  |  |  |
| Infants HUU | Ref |  |  |
| Infants HEU | -0.144 | -0.318 – 0.029 | 0.10 |
| **Maternal age** |  |  |  |
| 18-23 | Ref |  |  |
| 24-29 | -0.070 | -0.320 – 0.180 | 0.58 |
| 30-35 | -0.307 | -0.574 - -0.041 | 0.02 |
| 36-41 | -0.184 | -0.489 – 0.122 | 0.24 |
| 42-48 | -1.093 | -2.109 - -0.076 | 0.03 |
| **Employment status** |  |  |  |
| Not employed/ Not studying | Ref |  |  |
| Formal employment | 0.180 | -0.003 – 0.362 | 0.05 |
| Informal employment | 0.270 | -0.874 – 1.414 | 0.64 |
| Attending school/ College | -0.042 | -0.369 – 0.286 | 0.80 |
| Other, work or studying | 0.509 | -0.946 – 1.964 | 0.49 |
| **Marital/cohabiting status** |  |  |  |
| Not married/ Not cohabiting | Ref |  |  |
| Married/ Cohabiting | 0.294 | 0.112 – 0.458 | 0.001 |
| **Any breastfeeding** |  |  |  |
| No | Ref |  |  |
| Yes | 0.410 | -0.484 – 1.304 | 0.37 |
| **Household food security status** |  |  |  |
| Food insecure | Ref |  |  |
| Food secure | 0.173 | -0.108 – 0.454 | 0.23 |
| **Model 2: LAZ between infants HEU and HUU for any breastfeeding (N of observations = 2111)** | | | |
| **Effect** | **Estimate** | **(95% CI)** | ***p-*value** |
| **Visits** |  |  |  |
| 7 days | Ref |  |  |
| 10 weeks | -1.548 | -1.697 - -1.400 | < 0.001 |
| 6 months | -1.381 | -1.530 – -1.232 | < 0.001 |
| 12 months | -1.261 | -1.407 - -1.114 | < 0.001 |
| **Infant HIV exposure status** |  |  |  |
| Infants HUU | Ref |  |  |
| Infants HEU | -0.128 | -0.313 – 0.057 | 0.17 |
| **Maternal age** |  |  |  |
| 18-23 | Ref |  |  |
| 24-29 | 0.089 | -0.180 – 0.359 | 0.51 |
| 30-35 | -0.232 | -0.518 – 0.054 | 0.11 |
| 36-41 | -0.090 | -0.418 – 0.238 | 0.59 |
| 42-48 | -0.768 | -1.849 – 0.313 | 0.16 |
| **Employment status** |  |  |  |
| Not employed/ Not studying | Ref |  |  |
| Formal employment | -0.000 | -0.195 – 0.195 | 1.00 |
| Informal employment | 0.843 | -0.366 – 2.052 | 0.17 |
| Attending school/ College | 0.222 | -0.128- 0.572 | 0.21 |
| Other, work or studying | 0.629 | -0.954 – 2.212 | 0.43 |
| **Marital/cohabiting status** |  |  |  |
| Not married/ Not cohabiting | Ref |  |  |
| Married/ Cohabiting | 0.289 | 0.105 – 0.472 | 0.002 |
| **Any breastfeeding** |  |  |  |
| No | Ref |  |  |
| Yes | 0.217 | -0.727 – 1.162 | 0.65 |
| **Household food security status** |  |  |  |
| Food insecure | Ref |  |  |
| Food secure | -0.065 | -0.367 – 0.236 | 0.67 |
| **Model 3: WLZ between infants HEU and HUU for any breastfeeding (N of observations = 1525)** | | | |
| **Effect** | **Estimate** | **(95% CI)** | ***p-*value** |
| **Visits** |  |  |  |
| 10 weeks | Ref |  |  |
| 6 months | 0.125 | -0.022 – 0.272 | 0.09 |
| 12 months | 0.174 | 0.029 – 0.318 | 0.02 |
| **Infant HIV exposure status** |  |  |  |
| Infants HUU | Ref |  |  |
| Infants HEU | -0.007 | -0.197 – 0.184 | 0.95 |
| **Maternal age** |  |  |  |
| 18-23 | Ref |  |  |
| 24-29 | -0.356 | -0.634 - -0.078 | 0.01 |
| 30-35 | -0.340 | -0.635 - -0.046 | 0.02 |
| 36-41 | -0.226 | -0.563 – 0.110 | 0.19 |
| 42-48 | -0.791 | -1.889 – 0.307 | 0.16 |
| **Employment status** |  |  |  |
| Not employed/ Not studying | Ref |  |  |
| Formal employment | 0.163 | -0.038 – 0.364 | 0.11 |
| Informal employment | -0.647 | -1.863 – 0.570 | 0.30 |
| Attending school/ College | -0.206 | -0.567 – 0.155 | 0.26 |
| Other, work or studying | 0.524 | -1.166 – 2.214 | 0.54 |
| **Marital/cohabiting status** |  |  |  |
| Not married/ Not cohabiting |  |  |  |
| Married/ Cohabiting | 0.157 | -0.031 – 0.346 | 0.10 |
| **Any breastfeeding** |  |  |  |
| No | Ref |  |  |
| Yes | 0.524 | -0.442 – 1.490 | 0.29 |
| **Household food security status** |  |  |  |
| Food insecure | Ref |  |  |
| Food secure | 0.339 | 0.027 – 0.652 | 0.03 |

*LAZ* Length-for-age z-score, *WAZ* Weight-for-age z-score, *WLZ* Weight-for-length z-score,

*HEU* HIV-exposed-uninfected, *HUU* HIV-unexposed uninfected

| **Supplementary Table 4. Linear mixed effects models of WAZ, LAZ and WLZ between infants HEU and HUU**  **for exclusive breastfeeding up to 6 months**  **Model 1: WAZ between infants HEU and HUU for exclusive breastfeeding up to 6 months (N of observations = 2042)** | | | |
| --- | --- | --- | --- |
| **Effect** | **Estimate** | **(95% CI)** | ***p-*value** |
| **Visits** |  |  |  |
| 7 days | Ref |  |  |
| 10 weeks | -0.060 | -0.162 – 0.041 | 0.24 |
| 6 months | 0.366 | 0.265 – 0.467 | < 0.001 |
| 12 months | 0.626 | 0.525 – 0.727 | < 0.001 |
| **Infant HIV exposure status** |  |  |  |
| Infants HUU | Ref |  |  |
| Infants HEU | -0.132 | -0.314 – 0.509 | 0.16 |
| **Maternal age** |  |  |  |
| 18-23 | Ref |  |  |
| 24-29 | -0.103 | -0.367- 0.161 | 0.44 |
| 30-35 | -0.335 | -0.617 - -0.054 | 0.02 |
| 36-41 | -0.192 | -0.514 – 0.129 | 0.24 |
| 42-48 | -1.114 | -2.152 - -0.075 | 0.03 |
| **Employment status** |  |  |  |
| Not employed/ Not studying | Ref |  |  |
| Formal employment | 0.185 | -0.008 – 0.378 | 0.06 |
| Informal employment | 0.278 | -0.889 – 1.443 | 0.64 |
| Attending school/ College | -0.033 | -0.375 – 0.309 | 0.85 |
| Other, work or studying | -0.152 | -2.178 – 1.874 | 0.88 |
| **Marital/cohabiting status** |  |  |  |
| Not married/ Not cohabiting | Ref |  |  |
| Married/ Cohabiting | 0.310 | 0.129 – 0.491 | < 0.001 |
| **Exclusive breastfeeding up to 6 months** |  |  |  |
| No | Ref |  |  |
| Yes | -0.054 | -0.372 – 0.264 | 0.74 |
| **Household food security status** |  |  |  |
| Food insecure | Ref |  |  |
| Food secure | 0.251 | -0.046 – 0.548 | 0.10 |
| **Model 2: LAZ between infants HEU and HUU for exclusive breastfeeding up to 6 months (N of observations = 2016)** | | | |
| **Effect** | **Estimate** | **(95% CI)** | ***p-*value** |
| **Visits** |  |  |  |
| 7 days | Ref |  |  |
| 10 weeks | -1.508 | -1.660 - -1.355 | < 0.001 |
| 6 months | -1.364 | -1.515 - -1.212 | < 0.001 |
| 12 months | -1.217 | -1.369 - -1.065 | < 0.001 |
| **Infant HIV exposure status** |  |  |  |
| Infants HUU | Ref |  |  |
| Infants HEU | -0.125 | -0.317 – 0.067 | 0.20 |
| **Maternal age** |  |  |  |
| 18-23 | Ref |  |  |
| 24-29 | 0.047 | -0.234 – 0.326 | 0.74 |
| 30-35 | -0.226 | -0.523 – 0.071 | 0.14 |
| 36-41 | -0.067 | -0.407 – 0.273 | 0.70 |
| 42-48 | -0.732 | -1.823 – 0.359 | 0.19 |
| **Employment status** |  |  |  |
| Not employed/ Not studying | Ref |  |  |
| Formal employment | 0.002 | -0.201 – 0.206 | 0.98 |
| Informal employment | 0.880 | -0.337 – 2.098 | 0.16 |
| Attending school/ College | 0.201 | -0.160 – 0.561 | 0.27 |
| Other, work or studying | -0.439 | -2.555 – 1.677 | 0.68 |
| **Marital/cohabiting status** |  |  |  |
| Not married/ Not cohabiting | Ref |  |  |
| Married/ Cohabiting | 0.293 | 0.102 – 0.484 | 0.003 |
| **Exclusive breastfeeding up to 6 months** |  |  |  |
| No | Ref |  |  |
| Yes | -0.223 | -0.557 – 0.111 | 0.19 |
| **Household food security** |  |  |  |
| Food insecure | Ref |  |  |
| Food secure | 0.050 | -0.264 – 0.364 | 0.75 |
| **Model 3: WLZ between infants HEU and HUU for exclusive breastfeeding up to 6 months (N of observations = 1472)** | | | |
| **Effect** | **Estimate** | **(95% CI)** | ***p-*value** |
| **Visits** |  |  |  |
| 10 weeks | Ref |  |  |
| 6 months | 0.136 | -0.012 – 0.284 | 0.07 |
| 12 months | 0.175 | 0.027 – 0.323 | 0.02 |
| **Infant HIV exposure status** |  |  |  |
| Infants HUU | Ref |  |  |
| Infants HEU | 0.010 | -0.186 – 0.205 | 0.92 |
| **Maternal age** |  |  |  |
| 18-23 | Ref |  |  |
| 24-29 | -0.407 | -0.693 - -0.122 | 0.01 |
| 30-35 | -0.415 | -0.718 - -0.112 | 0.01 |
| 36-41 | -0.280 | -0.625 – 0.065 | 0.11 |
| 42-48 | -0.890 | -1.996 – 0.215 | 0.11 |
| **Employment status** |  |  |  |
| Not employed/ Not studying | Ref |  |  |
| Formal employment | 0.181 | -0.027 – 0.388 | 0.09 |
| Informal employment | -0.680 | -1.903 – 0.541 | 0.27 |
| Attending school/ College | -0.238 | -0.607 – 0.132 | 0.21 |
| Other, work or studying | -0.140 | -2.265 – 1.984 | 0.90 |
| **Marital/cohabiting status** |  |  |  |
| Not married/ Not cohabiting | Ref |  |  |
| Married/ Cohabiting | 0.189 | -0.005 – 0.383 | 0.06 |
| **Exclusive breastfeeding up to 6 months** |  |  |  |
| No | Ref |  |  |
| Yes | 0.184 | -0.152 – 0.520 | 0.28 |
| **Household food security status** |  |  |  |
| Food insecure | Ref |  |  |
| Food secure | 0.376 | 0.054 – 0.697 | 0.02 |

*LAZ* Length-for-age z-score, *WAZ* Weight-for-age z-score, *WLZ* Weight-for-length z-score,

*HEU* HIV-exposed-uninfected, *HUU* HIV-unexposed uninfected

| **Supplementary Table 5. Linear mixed effects models of WAZ, LAZ and WLZ between infants HEU and HUU (univariable analysis)**  **Model 1: WAZ between infants HEU and HUU (N of observations = 2661)** | | | | | |
| --- | --- | --- | --- | --- | --- |
| **Effect** | **Estimate** | | | **(95% CI)** | ***p-*value** |
| **Visits** |  | | |  |  |
| 7 days | Ref | | |  |  |
| 10 weeks | -0.125 | | | -0.216 - -0.034 | 0.007 |
| 6 months | 0.335 | | | 0.242 – 0.428 | < 0.001 |
| 12 months | 0.587 | | | 0.494 – 0.679 | < 0.001 |
| **Infant HIV exposure status** |  | | |  |  |
| Infants HUU | Ref | | |  |  |
| Infants HEU | -0.237 | | | -0.378 – -0.095 | 0.001 |
| **Model 2: LAZ between infants HEU and HUU (N of observations = 2618)** | | | | | |
| **Effect** | | **Estimate** | **(95% CI)** | | ***p-*value** |
| **Visits** | |  |  | |  |
| 7 days | | Ref |  | |  |
| 10 weeks | | -1.504 | -1.639 - -1.369 | | < 0.001 |
| 6 months | | -1.350 | -1.486 - -1.213 | | < 0.001 |
| 12 months | | -1.232 | -1.369 - -1.096 | | < 0.001 |
| **Infant HIV exposure status** | |  |  | |  |
| Infants HUU | | Ref |  | |  |
| Infants HEU | | -0.265 | -0.422 – -0.108 | | 0.0009 |
| **Model 3: WLZ between infants HEU and HUU (N of observations = 1825)** | | | | | |
| **Effect** | | **Estimate** | **(95% CI)** | | ***p-*value** |
| **Visits** | |  |  | |  |
| 10 weeks | | Ref |  | |  |
| 6 months | | 0.182 | 0.047 – 0.318 | | 0.008 |
| 12 months | | 0.213 | 0.076 – 0.350 | | 0.002 |
| **Infant HIV exposure status** | |  |  | |  |
| Infants HUU | | Ref |  | |  |
| Infants HEU | | -0.094 | -0.256 – 0.069 | | 0.26 |

*LAZ* Length-for-age z-score, *WAZ* Weight-for-age z-score, *WLZ* Weight-for-length z-score,

*HEU* HIV-exposed-uninfected, *HUU* HIV-unexposed uninfected

| **Supplementary Table 6. Linear mixed effects models of WAZ, LAZ and WLZ between infants HEU and HUU for duration of breastfeeding (excluding HFSS)**  **Model 1: WAZ between infants HEU and HUU for duration of breastfeeding (N of observations = 2397**) | | | | | |
| --- | --- | --- | --- | --- | --- |
| **Effect** | **Estimate** | | | **(95% CI)** | ***p-*value** |
| **Visits** |  | | |  |  |
| 7 days | Ref | | |  |  |
| 10 weeks | -0.071 | | | -0.165 - 0.022 | 0.14 |
| 6 months | 0.375 | | | 0.279 – 0.470 | < 0.001 |
| 12 months | 0.622 | | | 0.525 – 0.719 | < 0.001 |
| **Infant HIV exposure status** |  | | |  |  |
| Infants HUU | Ref | | |  |  |
| Infants HEU | -0.186 | | | -0.349 - -0.024 | 0.02 |
| **Maternal age** |  | | |  |  |
| 18-23 | Ref | | |  |  |
| 24-29 | 0.028 | | | -0.200 – 0.255 | 0.81 |
| 30-35 | -0.165 | | | -0.408 – 0.079 | 0.18 |
| 36-41 | -0.094 | | | -0.372 – 0.185 | 0.51 |
| 42-48 | -0.618 | | | -1.450 - 0.214 | 0.14 |
| **Employment status** |  | | |  |  |
| Not employed/ Not studying | Ref | | |  |  |
| Formal employment | 0.150 | | | -0.015 – 0.316 | 0.07 |
| Informal employment | 0.172 | | | -0.809 – 1.529 | 0.73 |
| Attending school/ College | 0.001 | | | -0.299 – 0.301 | 0.99 |
| Other, work or studying | 0.634 | | | -0.801 – 2.069 | 0.38 |
| **Marital/cohabiting status** |  | | |  |  |
| Not married/Not cohabiting | Ref | | |  |  |
| Married/ Cohabiting | 0.261 | | | 0.105 – 0.417 | 0.001 |
| **Duration of breastfeeding** | -0.000 | | | -0.000 – 0.000 | 0.99 |
| **Model 2: LAZ between infants HEU and HUU for duration of breastfeeding (N of observations = 2364)** | | | | | |
| **Effect** | | **Estimate** | **(95% CI)** | | ***p-*value** |
| **Visits** | |  |  | |  |
| 7 days | | Ref |  | |  |
| 10 weeks | | -1.520 | -1.659 - -1.380 | | < 0.001 |
| 6 months | | -1.360 | -1.502 - -1.218 | | < 0.001 |
| 12 months | | -1.240 | -1.387 - -1.099 | | < 0.001 |
| **Infant HIV exposure status** | |  |  | |  |
| Infants HUU | | Ref |  | |  |
| Infants HEU | | -0.202 | -0.381 – -0.022 | | 0.03 |
| **Maternal age** | |  |  | |  |
| 18-23 | | Ref |  | |  |
| 24-29 | | 0.107 | -0.146 – 0.360 | | 0.41 |
| 30-35 | | -0.156 | -0.426 – 0.114 | | 0.26 |
| 36-41 | | -0.023 | -0.331 – 0.285 | | 0.88 |
| 42-48 | | -0.448 | -1.364 – 0.469 | | 0.34 |
| **Employment status** | |  |  | |  |
| Not employed/ Not studying | | Ref |  | |  |
| Formal employment | | 0.031 | -0.152 – 0.214 | | 0.74 |
| Informal employment | | 0.971 | -0.097 – 2.038 | | 0.07 |
| Attending school/ College | | 0.181 | -0.149 – 0.511 | | 0.28 |
| Other, work or studying | | 0.733 | -0.863 – 2.330 | | 0.37 |
| **Marital/cohabiting status** | |  |  | |  |
| Not married/Not cohabiting | | Ref |  | |  |
| Married/ Cohabiting | | 0.220 | 0.048 – 0.392 | | 0.01 |
| **Duration of breastfeeding** | | -0.000 | -0.001 – 0.000 | | 0.44 |
| **Model 3: WLZ between infants HEU and HUU for duration of breastfeeding (N of observations = 1665)** | | | | | |
| **Effect** | | **Estimate** | **(95% CI)** | | ***p-*value** |
| **Visits** | |  |  | |  |
| 10 weeks | | Ref |  | |  |
| 6 months | | 0.157 | 0.018 – 0.297 | | 0.03 |
| 12 months | | 0.187 | 0.045 – 0.329 | | 0.01 |
| **Infant HIV exposure status** | |  |  | |  |
| Infants HUU | | Ref |  | |  |
| Infants HEU | | -0.015 | -0.202 – 0.171 | | 0.87 |
| **Maternal age** | |  |  | |  |
| 18-23 | | Ref |  | |  |
| 24-29 | | -0.303 | -0.565 - -0.041 | | 0.02 |
| 30-35 | | -0.289 | -0.568 - -0.011 | | 0.04 |
| 36-41 | | -0.212 | -0.528 – 0.104 | | 0.19 |
| 42-48 | | -0.614 | -1.556 – 0.329 | | 0.20 |
| **Employment status** | |  |  | |  |
| Not employed/ Not studying | | Ref |  | |  |
| Formal employment | | 0.150 | -0.039 – 0.340 | | 0.12 |
| Informal employment | | -0.968 | -2.042 – 0.107 | | 0.08 |
| Attending school/ College | | -0.144 | -0.486 – 0.198 | | 0.41 |
| Other, work or studying | | 0.647 | -1.040 – 2.334 | | 0.45 |
| **Marital/cohabiting status** | |  |  | |  |
| Not married/ Not cohabiting | | Ref |  | |  |
| Married/ Cohabiting | | 0.158 | -0.019 – 0.335 | | 0.08 |
| **Duration of breastfeeding** | | 0.000 | -0.000 – 0.000 | | 0.27 |

*LAZ* Length-for-age z-score, *WAZ* Weight-for-age z-score, *WLZ* Weight-for-length z-score,

*HEU* HIV-exposed-uninfected, *HUU* HIV-unexposed uninfected, *HFSS* Household Food Security Status
